# Functional genomics of mountain pine beetle (*Dendroctonus ponderosae*) midguts and fat bodies

**DOI:** 10.1186/1471-2164-11-215

**Published:** 2010-03-30

**Authors:** Tidiane Aw, Karen Schlauch, Christopher I Keeling, Sharon Young, Jeremy C Bearfield, Gary J Blomquist, Claus Tittiger

**Affiliations:** 1 Department of Biochemistry and Molecular Biology, University of Nevada, Reno, Reno, NV, 89557, USA; 2 Center for Bioinformatics, University of Nevada, Reno, Reno, NV, 89557, USA; 3 Current address: Michael Smith Laboratories, University of British Columbia, Vancouver, B.C., V6T 1Z4, Canada

## Abstract

**Background:**

The mountain pine beetle (*Dendroctonus ponderosae*) is a significant coniferous forest pest in western North America. It relies on aggregation pheromones to colonize hosts. Its three major pheromone components, *trans*-verbenol, *exo*-brevicomin, and frontalin, are thought to arise via different metabolic pathways, but the enzymes involved have not been identified or characterized. We produced ESTs from male and female midguts and associated fat bodies and used custom oligonucleotide microarrays to study gene expression patterns and thereby made preliminary identification of pheromone-biosynthetic genes.

**Results:**

Clones from two un-normalized cDNA libraries were directionally sequenced from the 5' end to yield 11,775 ESTs following sequence cleansing. The average read length was 550 nt. The ESTs clustered into 1,201 contigs and 2,833 singlets (4,034 tentative unique genes). The ESTs are broadly distributed among GO functional groups, suggesting they reflect a broad spectrum of the transcriptome. Among the most represented genes are representatives of sugar-digesting enzymes and members of an apparently Scolytid-specific gene family of unknown function. Custom NimbleGen 4-plex arrays representing the 4,034 tentative unique genes were queried with RNA from eleven different biological states representing larvae, pupae, and midguts and associated fat bodies of unfed or fed adults. Quantitative (Real-Time) RT-PCR (qRT-PCR) experiments confirmed that the microarray data accurately reflect expression levels in the different samples. Candidate genes encoding enzymes involved in terminal steps of biosynthetic pathways for *exo*-brevicomin and frontalin were tentatively identified.

**Conclusions:**

These EST and microarray data are the first publicly-available functional genomics resources for this devastating forestry pest.

## Background

**Figure 1:**
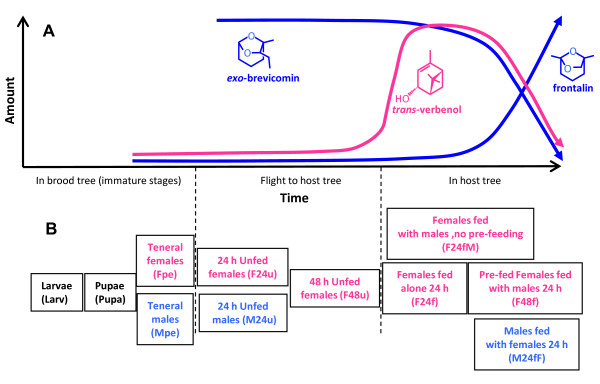
**Mountain pine beetle pheromone production and experimental samples**. (A) Developmental profile showing production of three important pheromone components by males (blue) and females (pink). Curves are based on data from Pureswaren *et al*. (2000). (B) The eleven biological conditions sampled for microarray analysis. Abbreviated labels are in parentheses.

The mountain pine beetle (*Dendroctonus ponderosae *Hopkins) is a major forest pest currently enjoying historically unprecedented outbreak populations in western North America [1]. Its success is due in part to the use of pheromones to coordinate mass-attacks on host trees, a behavior necessary to overcome tree oleoresin defences [2]. The aggregation pheromone system involves male- and female-produced components as well as synergistic effects of host monoterpenes [3,4]. Three important pheromone components are *trans*-verbenol [(1*S*, 2*R*, 5*S*)-4,7,7-trimethylbicyclo[3.1.1]hept-3-en-2-ol], *exo*-brevicomin (*exo*-7-ethyl-5-methyl-6,8-dioxabicyclo [3.2.1]octane), and frontalin [(1*S*, 5*R*)-1,5-dimethyl-6,8-dioxabicyclo[3.2.1]octane]. *trans*-Verbenol is produced by pioneer females when they attack a new host, serving as an aggregation pheromone that attracts males and other females to the tree. *exo*-Brevicomin is produced by males before they join females in the tree, serving as a synergist to *trans*-verbenol. Production of both *trans*-verbenol and *exo*-brevicomin falls later during colonization, at approximately the same time that males produce frontalin, which appears to function as an anti-aggregation or dispersion signal [4] (Figure 1A).

Understanding the metabolic pathways, their regulation, and the enzymes responsible for creating these chemicals is important in developing potentially targeted management strategies for this devastating forest pest and other destructive *Dendroctonus *spp. including the spruce beetle (*D. rufipennis*) and the southern pine beetle (*D. frontalis*). The three chemicals appear to arise via different metabolic pathways: *trans*-verbenol is likely the cytochrome P450-mediated hydroxylation product of ingested host tree α-pinene [5,6]; *exo*-brevicomin is produced from a fatty acyl-derived precursor [7,8]; and frontalin is synthesized *de novo *via the mevalonate pathway [9]. While common enzymes synthesizing their precursors are relatively easily identified, those creating the final pheromone components are more difficult to recognize.

Fortunately, advances in genomic and proteomic technologies allow creation of sequence and/or gene expression databases to investigate economically important but genetically inconvenient "non-model" species. For example, functional genomics studies of the pine engraver beetle, *Ips pini *(Coleoptera, Scolytidae) [10-12], assisted the identification of important pheromone-biosynthetic genes that otherwise would have been difficult to discover [13-15] and led to subsequent understanding of their regulation and biochemical roles [[13,16,17], Figueroa Teran *et al*., unpublished]. Similarly, a recent effort to characterize the proteome of *D. frontalis *pronota catalogued differences between males and females that may lead to new control measures [18]. We wished to create functional genomics tools for *D. ponderosae *to facilitate the development of new strategies to mitigate its economic impact. We produced ESTs representing over 4,000 tentative unique genes, and used custom oligonucleotide arrays to study their regulation in eleven different biological states. We have used these data to make preliminary identification of putative pheromone-biosynthetic genes. Furthermore, the ESTs and microarrays are resources for forest insect researchers working to understand bark beetles.

## Results and Discussion

### ESTs

Two cDNA libraries, "MPB" and "DPG," were prepared in pDONR222 (Invitrogen). The MPB library includes cDNAs from midguts and fat bodies of juvenile hormone (JH) III-treated and acetone-treated (control) insects because of the known role for JH III in stimulating pheromone biosynthesis in the Coleoptera [19], while the insects used for the DPG library were fed or unfed, but not stimulated with applied hormone and thus can be expected to have more normal biological expression patterns. The MPB library was constructed from 4.9 μg poly(A)+RNA, yielding a primary titre of 1.4 × 10^6 ^colony forming units (cfu). The average insert size, not including flanking vector sequences, was 1.3 ± 0.1 kb. The DPG library was constructed from 1.6 μg poly(A)+RNA, yielding a primary titre of 1.27 × 10^7 ^cfu and an average insert size of 1.2 ± 0.2 kb. In total, 2,867 and 9,594 (12,461 total) templates were sequenced from the MPB and DPG libraries respectively, yielding 12,119 total ESTs that passed the sequencer's quality control threshold (2,776 from MPB and 9,297 from DPG). The average read length for both libraries was 550 nt.

Further cleansing with EGAssembler [20] removed 344 additional ESTs. The remaining 11,775 ESTs were masked to remove vector and repeat sequences and clustered with CAP3 to yield 1,201 contigs and 2,833 singlets representing 4,034 tentative unique genes (TUGs). Of these, 2,040 returned at least one hit when compared against GenBank nr using BlastX and an E-value minimum cutoff of 10^-5^. The ESTs and assembled contigs have been deposited in GenBank [GO484341-GO495894] and [EZ114957-EZ116155], respectively.

**Figure 2:**
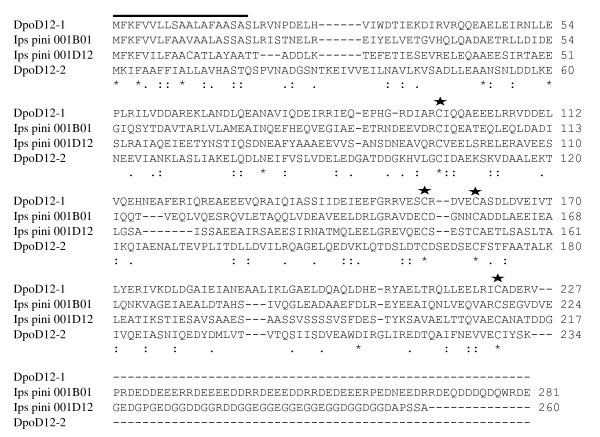
**ClustalW alignment of predicted translation products of a bark beetle-specific gene cluster**. The predicted protein sequences are for IPG001B01 [CB407747] and IPG001D12 [CB408591] from *I. pini *and DpoD12-1 [EZ115190] and DpoD12-2 [EZ115588] from *D. ponderosae*. Perfectly conserved amino acids are indicated by asterisks, similar amino acids are indicated by a colon below the alignment. The putative N-terminal signal peptide region is overlined. The conserved cysteine residues are indicated with a star above the alignment.

The nine contigs with the highest number of ESTs represent either sugar-digesting enzymes or orthologs of a bark beetle-specific gene cluster, *IPG001B01*/*IPG001D12*, identified in a previous EST survey of *Ips pini *midguts (Table 1) [10]. The abundance of sugar-degrading sequences reflects the midgut's digestive role and is consistent with EST surveys of other phytophagous beetles including *I. pini *[10], *Phaedon cochlearidae *[21], and *Chrysomela tremulae *[22]. The orthologs to *I. pini IPG001B1/001D12*, named here *DpoD12-1 *[GenBank: EZ115567] and *DpoD12-2 *[EZ115588], have essentially no sequence identity (4.6%) beyond a well-conserved N-terminal signal peptide, and lack the highly charged C-terminal tails of the *I. pini *proteins (Figure 2). However, all orthologs have four perfectly-conserved cysteines (Figure 2) and are predicted to have >95% α-helical structure (not shown). *DpoD12-1 *and *DpoD12-2*, which showed essentially stable, constitutively high expression in all samples based on microarray data (not shown), apparently contributed ~5.9% of recovered mRNA, as indicated by their portion of total ESTs, making them among the most highly-expressed genes sequenced. This is a much smaller though still significant fraction of the total than the ~35% observed for the *I. pini *orthologs [10]. The apparent difference in transcript abundance between species may reflect either a true difference in relative abundance, dilution of the midgut transcriptome by fat body mRNAs, or both. The highly-conserved secondary structures of these orthologs suggest conserved functions, which have not yet been determined.

**Table 1:** Genes with the highest representation by number of ESTs

Contig	GenBank I.D.	#ESTs	BlastX hit or identity	E-val	Species
Contig527	EZ115483	180	glycoside hydrolase	1.00e-139	*Tribolium castaneum*
Contig137	EZ115093	178	pectin esterase	1.66e-123	*Sitophilus oryzae*
Contig588	EZ115544	168	DpoD12-1	n.a.	*Ips pini*
Contig611	EZ115567	134	DpoD12-1	n.a.	*Ips pini*
Contig234	EZ115190	130	DpoD12-1	n.a.	*Ips pini*
Contig632	EZ115588	128	DpoD12-2	n.a.	*Ips pini*
Contig423	EZ115379	124	DpoD12-1	n.a.	*Ips pini*
Contig450	EZ115406	107	cello-biosidase	<1.00e-200	*Otiorhynchus sulcatus*
Contig841	EZ115796	103	β-endoglucanase	1.11e-54	*Apriona germari*
Contig467	EZ115423	98	aldo-keto reductase	1.19e-95	*Tribolium castaneum*

**Figure 3:**
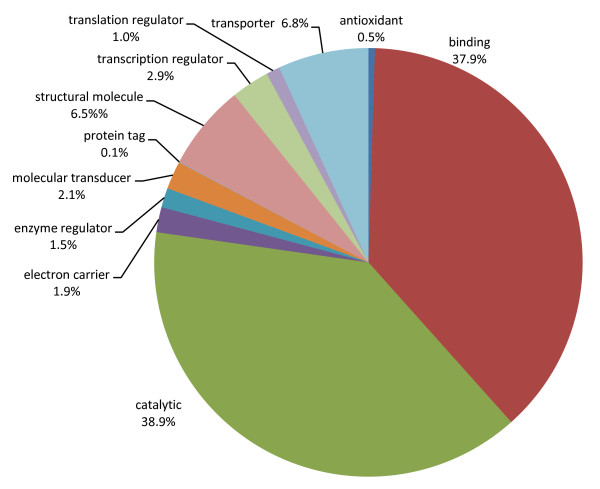
**Summary of gene annotations**. The relative proportions of genes based on molecular function GO terms at Level 2.

Representatives of other midgut-associated transcripts were sequenced, including those implicated in peritrophic membrane maintenance (chitinases, chitin deacetylase, chitin synthase, and chitobiosyldiphosphodolicol β-mannosidase), proteolysis (various proteases) and defence (cytochromes P450 and glutathione-S-transferases). Genes expressed in the fat body in other insects were also represented, including putative vitellogenin (DPG001D02), putative ferritin (contig638), and putative ommochrome-binding protein (DPG027E14). Gene ontology and enzyme number annotations show that a broad range of molecular functions were represented, with a preponderance of TUGs involved in catalysis or binding (Figure 3). The distribution is consistent with other samples of midgut transcriptomes [e.g. [10,22]] and is evidence that a broad spectrum of the midgut/fat body transcriptome was sampled.

### Microarrays

A major goal of this study was to combine sequence and microarray expression data to help identify pheromone biosynthetic and potential resin detoxification genes. To this end, we chose 11 biological states that spanned the beetle's life history and pheromone component profile (Figure 1B) to query custom oligonucleotide microarrays representing the 4,034 TUGs. Four biological replicate pools were generated for each of the 11 states. Visual inspection of all 44 hybridizations showed no gross spatial variation due to fibers or bubbles. The normalized distributions of expression values for all 44 arrays were very similar, with no apparent outlying arrays. Digestion curves suggested that trends in RNA degradation between 5' and 3' ends of each probe set were very similar. Thus we concluded that the quality of the arrays and the RNA used to query them was high. The array data have been deposited in NCBI [GEO: GPL9118, Sample ID numbers GSM446276 - GSM446319].

**Figure 4:**
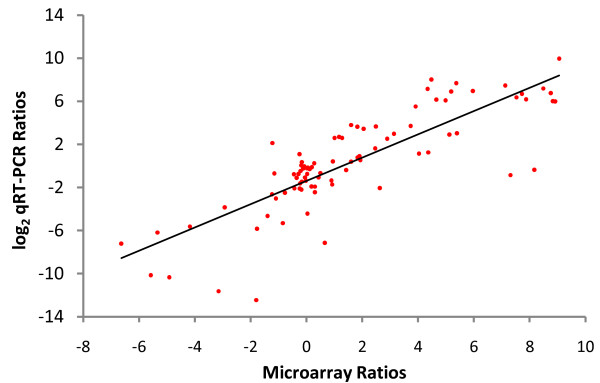
**Comparison of gene expression ratios for qRT-PCR and microarray data**. Nine genes (see Table 1) were selected for comparison. Relative expression values, normalized to *Ubiquitin*, for ten biological states were compared to those for unfed females (F24u) by qRT-PCR. The ratios of log_2_-transformed qRT-PCR data (*y*-axis) are plotted against the same ratios obtained from the microarray (*x*-axis). Linear regression showed a correlation coefficient of 0.825. More details are available in Additional file 1: Table S1.

To validate the array data, nine genes including mostly P450s and mevalonate pathway genes (Table 2) were chosen for quantitative (Real-Time) RT-PCR (qRT-PCR) amplification of first strand cDNA prepared from the same biological states used for microarray hybridizations. Linear regression of 90 pairs of qRT-PCR and microarray data showed an overall correlation coefficient of 0.825 (*P *<< 0.001) (Figure 4, Additional file 1: Table S1). Thus, there was a statistically significant strong to moderate correlation of qRT-PCR data with the microarray data, confirming that microarray values reliably indicate expression information.

**Table 2:** Quantitative (Real-Time) RT-PCR information

Feature	Tentative I.D.	Primer pair (Forward/Reverse)	Amplicon Length (bp)	T_m _(°C)	Amplification Efficiency (%)
Contig1127	P450 (CYP6)	ACTTCCCGCTGGATACAGACAT/GGATAAGACATCGTCTGGATTGTTG	101	76	90.91
Contig1103	P450 (CYP6)	AATGACTGCTTCGGTGCTGAA/ATTCCATGTCCCTACGATTGTGT	113	79	90.80
DPG001G12	P450 (CYP4)	CCGTAATCCCACAATGTTCGA/CCAAGGCAAAGTCTAGGTCCAT	117	78	92.41
Contig64	P450 (CYP6)	GCAAGAGGAATCAACCGCTAA/CTATGCTGCCTCAGCTCGTTATT	128	79	86.46
Contig608	P450 (CYP6CR1)	GAGGAACCACATAGTTGTCATGGA/CAAAAGGGAGGCGGATGTTA	141	73	93.3
Contig160	P450 (CYP6)	AATGATTGCTTCGGCACTGAA/GGATTTGAGTAATTCCATGCTCCTA	113	76	105.64
MPB029F09	P450 (CYP6)	ACTGGTAACGGACTACGATCACTTT/TGAACGCAATACTTTCCATTCG	115	75	89.16
MPB019E07	HMGR	CCAATCACCCGTGGGAAGT/CGAAGTGGAGGTTGCTGTTCA	82	81	95.45
Contig126	GGPPS	TGAACGTGCCCAAAGAGAATT/TCGGCTAGTTTAGCTCGGATATTT	101	77	92.12

### Clustering

Genes active in the terminal steps of ipsdienol biosynthesis in *I. pini *share three common expression characteristics: they are induced in pheromone-biosynthetic tissues by juvenile hormone, their basal expression levels are higher in males than in females, and they are coordinately regulated with mevalonate pathway genes that function earlier in the pathway [11,12]. Combining these data allowed the identification of previously unknown pheromone-biosynthetic genes, including the dual-function geranyldiphosphate synthase/myrcene synthase [15,16], myrcene hydroxylase [14], and a novel oxidoreductase (Figueroa Teran *et al*., unpublished data). Therefore, we clustered the current microarray data to assist in the identification of *D. ponderosae *pheromone-biosynthetic genes.

**Figure 5:**
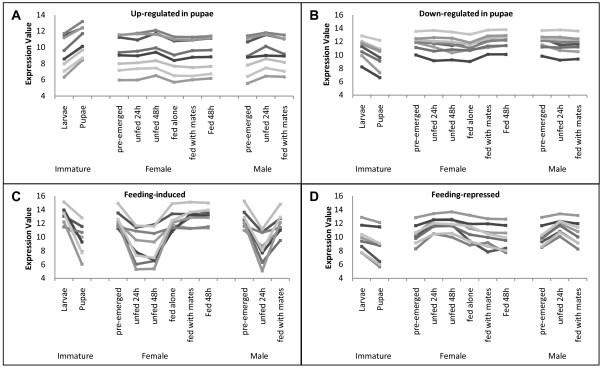
**Representative samples of gene clusters based on microarray data**. Several clusters included genes that are up-regulated (A) or down-regulated (B) in pupae, or induced (C) or repressed (D) by feeding. Lines connect expression levels for each gene but are not meant to imply a linear change between biological states. Expression values are processed, normalized, and log_2_-transformed microarray data for whole juvenile stages or midguts and fat bodies of adults. See Additional file 3: Table S3 for gene names.

Hierarchical clustering yielded 299 clusters of between 2 and 162 TUGs (2,485 features, total) with at least 0.85 average pairwise correlation amongst cluster member expression profiles across the 11 biological states (Additional file 2: Table S2). Visual inspection suggested that many clusters contained features with relatively stable expression across all treatment groups. Several clusters involved genes that were either up- or down-regulated in pupae (e.g. Figure 5) compared to other stages, consistent with the dramatic change in gene regulation, dietary intake, and metamorphosis occurring in developing pupae. Several clusters of genes that were either up- or down-regulated by feeding (e.g. Figure 5, Additional file 3: Table S3) were also apparent, as can be expected for midgut and fat body tissues that must respond to dietary status.

### Putative pheromone-biosynthetic genes

**Figure 6:**
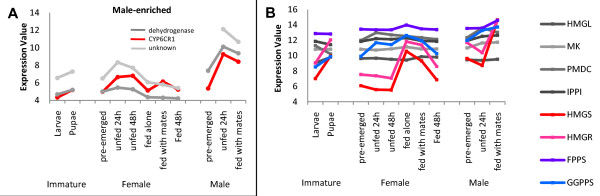
**Representative microarray clusters of genes with elevated expression in females or males**. Genes with these expression profiles may be involved in pheromone production. A "male-enriched" cluster (A) includes CYP6CR1 (red curve) and has an expression pattern consistent with *exo*-brevicomin production. (B) Mevalonate gene expression. The putative GGPPS representative (contig126, blue curve) has an expression profile similar to those for HMGS and HMGR. Expression values are processed, normalized, and log_2_-transformed microarray data for whole juvenile stages or midguts and fat bodies of adults. FPPS, farnesyl diphosphate synthase; GGPPS, geranylgeranyl diphosphate synthase; HMGL, HMG-CoA lyase; HMGS, HMG-CoA-synthase; HMG-R, HMG-CoA-reductase; IPPI, isopentenyl diphosphate isomerase; MK, mevalonate kinase; PMDC, phosphomevalonate decarboxylase.

*exo*-Brevicomin is thought to arise from a fatty-acyl precursor [7,8], implying that fatty acid-biosynthetic (and possible-catabolic) mRNAs should be elevated in unfed males. Fatty acid-metabolizing genes (e.g. acetyl-CoA carboxylase, acyl-carrier protein (ACP), ACP transferase, β-ketoacyl ACP reductase, desaturases, acetyl-CoA synthetase, enoyl hydratase, etc.) did not appear to be coordinately regulated or have uniformly higher basal expression levels in males compared to females (not shown). Similarly, mevalonate pathway genes (e.g. 3-hydroxy-3-methylglutaryl CoA (HMG) synthase (HMGS), HMGR, mevalonate kinase, diphosphomevalonate decarboxylase, isopentenyl diphosphate (IPP) isomerase, etc.) necessary for *de novo *frontalin production [9] were not clearly coordinately regulated (Figure 6B). There are various potential explanations for the apparent lack of coordinate gene regulation. The amount of frontalin recovered from volatile extracts of *D. ponderosae *(~90 ng/male) is much lower than that of ipsdienol from *I. pini *(~600 ng/male), so if the change of gene expression correlates with the amount of pheromone component, the induction may be too small to detect on microarrays.

**Figure 7:**
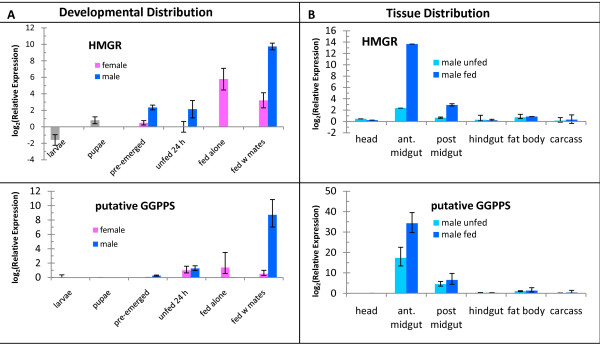
**qRT-PCR analysis of HMGR and putative GGPPS**. Normalized mRNA levels are shown relative to levels in unfed females with respect development (A) and relative to levels in heads of unfed insects for tissue distribution in males (B). Note that basal expression levels of putative GGPPS are much higher than those for HMGR.

Alternatively, the pheromone-biosynthetic pathway may be loosely coordinated. In *I. pini*, early pheromone-biosynthetic pathway genes have relatively lower basal expression levels and are much more strongly induced by JH III than genes functioning near the terminal steps, which have higher basal expression levels but are not as strongly induced [11]. Thus, genes for mevalonate or lipid-metabolizing pathways may not necessarily be closely coordinated. Furthermore, the microarrays were queried with RNA combined from midguts and fat bodies, so expression changes in one tissue may be masked by those of the other. In fact, mevalonate gene expression patterns appeared quite different in midguts and fat bodies (Figure 7 and not shown). HMGR and HMGS mRNA levels rise in female *Blattella germanica *fat bodies upon egg production [23], and a similar increase in mated female *D. ponderosae *may mask stable expression levels in midguts. Any combination of these scenarios is also possible. It is also possible that some pheromone-biosynthetic genes may have been missed due to the relatively small EST sample size.

The considerations noted above support the assertion by Keeling *et al. *[11] that microarray data alone are not reliable indicators of a gene's potential role. While the relatively small EST sample size confers a risk that some pheromone-biosynthetic genes were missed, the available data were still useful for preliminary identification of pheromone-biosynthetic genes, particularly those involved in later steps. For example, *exo*-brevicomin biosynthesis requires the fatty acyl-derived precursor, (*Z*)-6-nonen-2-one, to be epoxidized to 6,7-epoxynonan-2-one, a reaction that is likely catalyzed by a cytochrome P450 [8]. One cluster of three genes with a "male-enriched" expression profile contains a cytochrome P450 (CYP6CR1), a putative dehydrogenase, and an unknown protein (Figure 6A). Further qRT-PCR analyses indicate that the P450 gene has expression characteristics consistent with *exo*-brevicomin biosynthesis, suggesting that it may carry out the epoxidation step, while the dehydrogenase has characteristics implying a role in fatty-acyl-CoA metabolism upstream of epoxidation (G. Song *et al*., unpublished data). Similarly, frontalin biosynthesis requires carbon to be shunted from the mevalonate pathway either by GPPS (a short-chain isoprenyl diphosphate transferase) [15], or by a dioxygenase active on GPP or longer-chain isoprenoids, or by another, as yet unknown pathway. One gene, represented by contig126, has a best blastx hit to geranylgeranyl diphosphate synthase (GGPPS, a long chain isoprenyl diphosphate transferase) and an expression profile congruent with HMGR and HMGS (Figure 6B, Figure 7) and with frontalin production: it is induced by feeding, with most of the mRNA localizing to the midgut (Figure 7), where frontalin is produced [9]. While the expression profile is consistent with one expected of a putative GPPS, the activity of this enzyme must be determined. The lack of sequence similarity between this gene and *I. pini *GPPS may perhaps reflect both broad evolutionary divergence and potentially different enzyme activities. *I. pini *GPPS also functions as myrcene synthase [16], an activity that one would not expect in *D. ponderosae *because myrcene is not a predicted intermediate in frontalin biosynthesis. Both CYP6CR1 (putative epoxidase) and contig126 (putative GGPPS) are current subjects of post-genomic experiments to confirm their functions.

## Conclusion

We have generated EST and microarray tools for *D. ponderosae*. The ESTs and microarray data will be useful for researchers working to develop control strategies for this important pest insect. The microarray data suggest genes encoding enzymes in early steps of metabolic pathways implicated in pheromone component biosynthesis are not clearly coordinately regulated. However, candidate enzymes catalyzing late steps of *exo*-brevicomin and frontalin biosynthesis were identified, and are subjects of ongoing post-genomic research.

## Methods

### Insects and treatments

*Dendroctonus ponderosae *were obtained from infested lodgepole pine (*Pinus contorta*) in Little Valley, NV (39°15'00" N, 119°52'30"W). Infested trees were cut into 1-1.5 m long bolts and placed in rearing boxes as per [24]. Adults that emerged from the bolts were sexed and stored at 4°C on moist paper towels for up to two weeks. Larvae, pupae, and teneral adults were removed from beneath the bark and used immediately. For juvenile hormone (JH) treatment, 10 μg (±)JH III (Sigma) in 0.5 μl acetone was applied to the ventral abdomens of females or males (30 insects/group), followed by incubation in the dark at room temperature in 60 ml plastic containers for 8 or 16 hours. Control insects were treated with 0.5 μl acetone and incubated similarly. For fed samples, females or males were placed in ~4 mm holes drilled through the outer bark of *P. contorta *bolts and held there with wire mesh for the desired feeding period, after which the beetles were removed from under the bark. Unfed females or males were incubated in plastic cups in the dark for the same amount of time.

Beetles were chilled on ice and then dissected in distilled H_2_O to remove the midguts and associated fat bodies: in our hands it was virtually impossible to isolate midguts without some fat body remaining attached. The midgut/fat body tissues were pooled according to treatment group and stored at -84°C until use.

### cDNA library construction

Two cDNA libraries were prepared in pDONR222 (Invitrogen). The first, "MPB," was prepared using RNA isolated from midguts and fat bodies of eight treatment groups: females or males that had been unfed for 24 h, fed (each sex alone) for 24 h, or treated with JH III and incubated for 8 or 16 h. There were five sets of each treatment group, with 30 beetles/set. The second library, "DPG," was prepared from RNA isolated from midguts and fat bodies of six treatment groups: unfed females or males, females that had been fed alone for 6 or 24 h, females that had been fed alone for 24 h and then with males for 24 h ("fed with mates"), and males that were fed 24 h in the presence of females that had been feeding for 24 h. There were four replicates of 20 beetles/replicate for each condition. For both libraries, total RNA was isolated from each sample using RNeasy Plant Mini Kit (Qiagen). Poly(A)+RNA was isolated by twice passing the pooled total RNA over oligo-dT latex beads (Nucleotrap, Clontech) according to the manufacturer's protocol. cDNAs were prepared and directionally cloned into pDONR222 using the CloneMiner cDNA library construction kit (Invitrogen) and stored at -84°C until use. Insert sizes were determined by PCR amplification with vector primers across the inserts of 20 random clones for each library. Primary titres were determined by growth assays of serially-diluted library stocks.

### Sequencing and bioinformatics

A fraction of the MPB library was plated on LB agar containing 50 μg/ml kanamycin and 2,880 colonies were manually picked into 100 μl of TB-kanamycin broth in wells of 30 96-well plates and provided to the Nevada Genomics Center for incubation, plasmid preparation, and single-pass sequencing from the 5' ends using the M13 (Forward) vector primer. Sequencing reactions were performed using an ABI Prism 3730 DNA analyzer. For the DPG library, Agencourt Biosciences was contracted to prepare templates and Sanger sequence single-pass 5' reads of 10,000 individual colonies. The Sanger method was chosen for sequencing because it was the most effective technology available; pyrosequencing technology was not developed enough for our purposes at the time this study was done. The sequences from both libraries were trimmed of low confidence signals using the sequencers' software. The trimmed sequences were combined and further cleansed to remove vector sequences and short reads (<100 nt) using the EGAssembler website [20]. The cleansed ESTs were clustered using CAP3 [25] set with the following parameters: o = 40, p = 95, s = 401, e = 12. Contigs and singlets were annotated by BLASTX alignments to GenBank nr using Blast2GO freeware [26]. Multiple sequence alignments were done with the ClustalW2 [27] server at EBI using default settings.

### Microarrays

Roche NimbleGen was contracted to fabricate and hybridize 4-plex microarrays based on 4,034 *D. ponderosae *sequences from EST assembly and four negative control sequences: pDONR222, pBluescript, *Hevea brasiliensis *2-C-methyl-D-erythritol 2,4-cyclodiphosphate synthase [GenBank: AB294705], and *Pinus halepensis *partial mRNA for rubisco large subunit [GenBank: AJ271897]. Each feature was represented by six 60-mer probes distributed through the sequence, replicated in three non-contiguous blocks for each array.

The arrays were queried with cDNA prepared from 20-30 pooled insects representing 11 different biological states (Figure 1B): larvae (Larv), pupae (Pupa), teneral ("pre-emerged") females (Fpe) or males (Mpe), females that were unfed for 24 h (F24u) or 48 h (F48u), females that fed on *P. contorta *phloem for 24 h (F24f), females fed for 24 h alone and then with males for an additional 24 h (F48f), females that were fed with males for 24 h without "pre-feeding" alone (F24fM), males that were unfed for 24 h (M24u), and males that were allowed to feed for 24 h in the presence of females (M24fF). Total RNA was prepared from whole bodies of immature stages or from isolated midguts and fat bodies of adults as described above, treated with DNase, and precipitated at -84°C until use. The quality of RNA was confirmed with an Agilent 2100 Bioanalyzer and quantity was determined with a NanoDrop spectrometer (Thermo Scientific). Cy3-labeled cDNA was prepared from the RNA and hybridized to the arrays by Roche NimbleGen. Four biological replicates were prepared for each biological state, for a total of 44 hybridizations.

### Microarray data processing and analysis

All NimbleGen custom oligonucleotide array images were examined visually for gross spatial variation due to fibers or bubbles. All array data were processed and normalized first by Robust Multi-Array Average (RMA) [28] using the R package affy [29]. Specifically, expression values were computed by applying the RMA model of probe-specific correction of perfect match probes. The processed probe values were then normalized via quantile normalization, and a median polish was applied to compute one expression measure from all probe values. A visual inspection showed that the normalized distributions of expression values of all 44 arrays were very similar, with no apparent outlying arrays. Curves representing the trends in RNA degradation between the 5' end and the 3' end in each probeset were generated and inspected and all proved very similar.

To ensure strict reproducibility standards, the quadruplicated expression measurements for each biological state were inspected individually. Any set of quadruplicates in which one of the measures exhibited a standard deviation of more than 1.25 (the maximum possible standard deviation for four measures is 1.499), and a coefficient of variation of greater than 0.75 for the quadruplicate set was scrutinized. If one single measure was near 1.5, this indicated that the remaining three measures were very similar, and that the fourth replicate was at its maximum outlying capacity, and thus this one quadruplicate value was removed. This procedure left three replicates within the set of which the mean was used for subsequent analyses. Only 504 replicate sets (1.2% of all replicated sets) had one measure excluded by this rule. Additionally, any replicate sets that continued to exhibit a coefficient of variation of greater than 0.75 were removed entirely. This included only 235 sets of triplicated measures (0.5% of all replicate measurements), and reduced the mean coefficient of variation of all quadruplicates to 0.19. We found that these thresholds allowed us to identify gross outlying individual measurements within a quadruplicate set [30].

A simple 1-way ANOVA was performed on the normalized data to determine which features on the array were differentially expressed across the eleven states. A multiple testing correction was applied to the p-values of the ANOVA [31], and any feature with a significant adjusted p-value *P *< 0.05 was examined further with a Tukey's post-hoc test. Specifically, any feature that showed a significant difference in means across one of pairwise comparisons: M24u-M24fF, Mpe-M24u, Larv-F24u, M24u-Larv, M24fF-F24fM, F24fM-F24f, M24u-F24f, F24u-F24f, M24fF-Larv, Mpe-M24fF, Mpe-Fpe, F48f-F24f, F48u-F24f, Larv-F24fM, M24u-F24u was retained for further analysis.

The retained features (3,573 total) were then subjected to hierarchical clustering using Pearson's correlation coefficient as distance metric and the average agglomeration method. Clustering dendrograms were examined below the 0.15 height threshold, allowing a close inspection of genes clustered at or above a cluster-average correlation coefficient of 0.85.

Nine features for which preliminary qRT-PCR experiments showed clean amplification efficiencies and melting curves were used to confirm the microarray data. First strand cDNA was prepared and primer pairs were selected as described below. For each of the nine features, log_2_-transformed ratios of expression relative to unfed females (F24u, arbitrarily chosen as a reference sample) were determined for the qRT-PCR data, normalized to the endogenous control gene, *Ubiquitin*. The ten qRT-PCR ratios were compared to the analogous log-transformed ratios of the microarray data of the other ten biological states using Pearson's correlation coefficient. A linear regression of 10 pairs of log-transformed qRT-PCR/microarray expression ratios was performed for all nine genes (90 pairs, total). A hypothesis test was performed to evaluate the association between PCR and microarray data, using a test statistic based on Pearson's product moment correlation coefficient, and resulted in a p-value *P *<< 0.001. Steps for performing these verifications were identical to those in [32].

### Quantitative RT-PCR

To confirm the microarray data, first strand cDNA template was prepared from RNA isolated from combined midguts and fat bodies of adults, or whole bodies of immature stages using the RNeasy Plant Mini Kit (Qiagen) followed by RNase-free DNase digestion and further purification using the MasterPure RNA Purification kit (Epicentre). There were 20 insects/replicate, except for the pupal samples, for which six individuals were used. For the tissue distribution assays, beetles were dissected in water into head, anterior midgut, posterior midgut, hindgut, fat body (not associated with the anterior midgut), and carcass with the aid of a dissecting microscope essentially as per [14]. Tissues were pooled from five to eight beetles/sample, frozen in N_2_(l) and stored at -84°C prior to RNA extraction and purification. RNA pellets were washed with 70% ethanol, resuspended in TE, and checked for quantity and integrity as described above. First strand template cDNA was produced from 500 ng RNA for each sample using random primers (Invitrogen) and SuperScript III reverse transcriptase (Invitrogen) according to the manufacturer's protocol.

Primers for qRT-PCR were designed from the EST sequences using Primer Express v 2.0 software (Applied Biosystems). Selected primers (Table 2) were screened for potential primer-dimers and hairpin loop formation using Vector NTI Advance 9 (Invitrogen), and selected primer sets were tested for non-specific amplification by visual inspection of melting curves, and their amplification efficiencies were determined using a relative standard curve method. A survey of candidate normalizing genes using qBase [33] indicated *tubulin *and *Ubiquitin *[GenBank: EZ115790 and EZ115624, respectively] had the most stable expression across all samples (not shown). Relative expression values for all genes were determined using the ΔΔC_T _method [34] normalized to *tubulin *and *Ubiquitin*. There were four replicates of each biological sample, each containing material pooled from five to eight insects. There were two template preparations per sample with three technical replicates for each PCR reaction.

## Authors' contributions

CT and GJB conceived the design of the study. TA, CIK, JB and GJB obtained beetles. TA, CIK, JB, and SY prepared biological samples and isolated RNA. SY curated the EST clones, prepared samples for sequencing, and identified normalizing genes. CT constructed the libraries, analyzed the ESTs and prepared part of the manuscript. KS performed the microarray data quality control and clustering analyses. TA performed the qRT-PCR and prepared parts of the manuscript. All authors have read and approved the final manuscript.

## Supplementary Material

Additional file 1 **Table S1: Summary of qRT-PCR and microarray comparisons**. Relative expression values for nine selected genes (Features) in 10 different biological states, each compared to unfed females (F24u). The ratios were determined by qRT-PCR and from microarray data as described in the text. Values in this table were used to create Figure 4. Click here for file

Additional file 2 **Table S2: Cleansed, normalized and clustered microarray expression values**. Microarray expression values are provided for those genes (Features) with at least 0.85 average pairwise correlation across the 11 biological states to create clusters. GO identifiers are also provided. Click here for file

Additional file 3 **Table S3: Information for genes shown in Figure **5. Names, GenBank accession numbers, and tentative BLSTX identifications of genes (Features) in clusters incorporated into Figure 5. Click here for file
