# Supplementary material for: Functional genomics of mountain pine beetle (Dendroctonus ponderosae) midguts and fat bodies
Source: BMC Genomics. 2010 Mar 30;11:215. doi: 10.1186/1471-2164-11-215 (PMC2858752; doi:10.1186/1471-2164-11-215)
Supplement: Additional file 1 — Table S1: Summary of qRT-PCR and microarray comparisons. Relative expression values for nine selected genes (Features) in 10 different biological states, each compared to unfed females (F24u). The ratios were determined by qRT-PCR and from microarray data as described in the text. Values in this table were used to create Figure 4. [file 1471-2164-11-215-S1.PDF]

Table S1: Summary of qRT-PCR and Microarray Comparisons. Values are ratios with respect to F24u

qRT-PCR values[a]

Feature	DPG001G12	DPG004B18	DPG004O08	DPG010M04	DPG014B22	DPG015M04	MPB029F09	MPB GGPPS	MPB HMG-R
Larv	0.03	0.46	0.05	13.86	0.02	0.18	0.01	0.00	0.77
Pupa	5.75	0.00	0.00	0.03	0.07	2.13	6.47	0.00	2.38
Fpe	145.30	0.97	0.39	194.46	0.86	0.58	176.57	0.02	0.63
F24U	1.00	1.00	1.00	1.00	1.00	1.00	1.00	1.00	1.00
F48U	0.39	0.72	0.84	4.35	0.36	1.03	0.26	0.23	0.47
F24F	123.80	1.28	12.39	260.58	6.01	0.12	141.14	1.34	67.53
F24fM	0.78	0.92	0.24	7.50	1.71	0.62	0.55	1.19	13.04
F48F	64.18	0.81	3.07	119.56	1.88	0.16	72.77	0.04	6.03
Mpe	108.79	0.27	0.59	8.19	0.24	1.45	103.46	0.18	2.19
M24U	0.60	0.33	0.01	12.70	0.01	71.48	0.22	1.31	7.90
M24fF	63.62	0.89	0.48	205.45	0.30	45.71	82.16	10.91	996.89

Microarray ratio values[b]

Feature	DPG001G12	DPG004B18	DPG004O08	DPG010M04	DPG014B22	DPG015M04	MPB029F09	MPB GGPPS	MPB HMG-R
Larv	NA	0.78	1.03	3.04	0.06	0.59	1.58	0.11	2.69
Pupa	7.44	0.02	0.03	0.56	0.13	0.84	2.26	0.29	20.65
Fpe	359.53	0.95	1.86	NA	1.01	0.73	139.75	0.29	1.41
F24U	1.00	1.00	1.00	1.00	1.00	1.00	1.00	1.00	1.00
F48U	0.99	0.87	0.92	0.43	0.89	0.88	1.23	0.85	0.96
F24F	62.49	0.89	3.55	22.28	2.01	0.47	20.32	1.93	31.74
F24fM	287.34	1.14	6.19	34.92	3.57	0.45	158.99	1.21	13.32
F48F	456.34	1.08	5.52	36.40	3.73	0.43	234.59	0.38	2.43
Mpe	432.03	1.14	1.02	42.00	0.74	3.82	211.49	1.24	16.41
M24U	0.82	0.86	0.02	5.61	0.01	25.26	0.88	3.03	8.80
M24fF	483.15	1.04	1.35	41.34	1.90	15.06	184.84	4.14	534.49

a) Values are ($2^{\wedge}$-$\Delta\Delta C_T$) normalized to *Ubiquitin* as described in the text

b) Normalized and cleansed as described in the text
