# Supplementary material for: Functional genomics of mountain pine beetle (Dendroctonus ponderosae) midguts and fat bodies
Source: BMC Genomics. 2010 Mar 30;11:215. doi: 10.1186/1471-2164-11-215 (PMC2858752; doi:10.1186/1471-2164-11-215)
Supplement: Additional file 3 — Table S3: Information for genes shown in Figure 5. Names, GenBank accession numbers, and tentative BLSTX identifications of genes (Features) in clusters incorporated into Figure 5. [file 1471-2164-11-215-S3.PDF]

Table S3: Information for genes shown in Figure 5

Trend	Feature Name	Accession	BLASTX Tentative I.D.	E value	Species
Up-regulated in pupae					
	Contig959	EZ115913	ADP ribosylation factor	8e-56	*Tribolium castaneum*
	DPG005F15	GO488580	Nervous wreck	2e-35	*Apis mellifera*
	DPG007O13	GO489418	Nucleotide exchange factor	4e-15	*Tribolium castaneum*
	DPG017O22	GO492765	Ribosomal protein S3a	2e-78	*Tribolium castaneum*
	DPG019B22	GO493161	Plexin B	5e-06	*Tribolium castaneum*
	MPB003C05	GO484554	No significant hit		
	MPB007B05	GO484923	Hypothetical protein	2e-12	*Tribolium castaneum*
	MPB009E10	GO485149	No significant hit		
Down-regulated in pupae					
	Contig838	EZ115793	Similar to 10G08	2e-18	*Tribolium castaneum*
	Contig905	EZ115860	Cytochrome P450	2e-44	*Tribolium castaneum*
	Contig977	EZ115931	Myosin 1B	1e-58	*Tribolium castaneum*
	DPG001G19	GO487268	DnaK chaperone protein	1e-30	*Rhizobium* spp.
	DPG005E13	GO488555	Cytochrome P450	1e-50	*Tribolium castaneum*
	DPG009H16	GO489935	unknown		
	DPG016N01	GO492376	Short-chain dehydrogenase	8e-32	*Tribolium castaneum*
	DPG021M15	GO494092	Short-chain dehydrogenase	2e-46	*Tribolium castaneum*
Feeding induced					
	Contig354	EZ115310	No significant hit		
	Contig786	EZ115741	No significant hit		
	Contig948	EZ115902	esterase	2e-75	*Tribolium castaneum*
	Contig1176	EZ116130	Hypothetical protein	4e-12	*Tribolium castaneum*
	DPG015M16	GO492020	unknown		
	DPG018L06	GO493025	Peroxisomal membrane protein PMP22	1e-69	*Tribolium castaneum*
	DPG025G15	GO495020			
	MPB014D04	GO485599	Fat body protein	8e-35	*Tribolium castaneum*
Feeding repressed					
	Contig120	EZ115076	Monocarboxylate transporter	1e-51	*Tribolium castaneum*
	Contig322	EZ115278	No significant hit		

	Contig351	EZ115307	Tetraspanin	2e-23	*Tribolium castaneum*
	Contig773	EZ115728	No significant hit		
	DPG015E11	GO491847	Hexokinase	1e-37	*Tribolium castaneum*
	DPG024D06	GO494584	No significant alignment		
	MPB020G06	GO486199	Monocarboxylate transporter	1e-20	*Tribolium castaneum*
	MPB021E12	GO486277	AMP-dependent CoA ligase	7e-14	*Tribolium castaneum*
